# Fasting Hyperglycemia Increases In-Hospital Mortality Risk in Nondiabetic Female Patients with Acute Myocardial Infarction: A Retrospective Study

**DOI:** 10.1155/2014/745093

**Published:** 2014-07-14

**Authors:** Guojing Luo, Hong Liu, Shunkui Luo, Fang Li, Minhong Su, Hongyun Lu

**Affiliations:** Department of Endocrinology and Metabolism, the Fifth Affiliated Hospital of Sun Yat-sen University, Zhuhai, Guangdong 519000, China

## Abstract

Previous studies had shown that elevated admission plasma glucose (APG) could increase mortality rate and serious complications of acute myocardial infarction (AMI), but whether fasting plasma glucose (FPG) had the same role remains controversial. In this retrospective study, 253 cases of AMI patients were divided into diabetic (*n* = 87) and nondiabetic group (*n* = 166). Our results showed that: compared with the nondiabetic patients, diabetic patients had higher APG, FPG, higher plasma triglyceride, higher rates of painless AMI (*P* < 0.01), non-ST-segment elevation myocardial infarction (NSTEMI), and reinfraction (*P* < 0.05). They also had lower high density lipoprotein cholesterol and rate of malignant arrhythmia, but in-hospital mortality rate did not differ significantly (*P* > 0.05). While nondiabetic patients were subgrouped in terms of APG and FPG (cut points were 11.1 mmol/L and 7.0 mmol/L, resp.), the mortality rate had significant difference (*P* < 0.01), whereas glucose level lost significance in diabetic group. Multivariate logistic regression analysis showed that FPG (OR: 2.014; 95% confidence interval: 1.296–3.131; *p* < 0.01) but not APG was independent predictor of in-hospital mortality for nondiabetic patients. These results indicate that FPG can be an independent predictor for mortality in nondiabetic female patients with AMI.

## 1. Introduction

Incidence of AMI in female patients is increasing year by year after menopause, especially for type 2 diabetes mellitus (T2DM) patients. Gender disparity in clinical outcome of AMI patients with or without T2DM is still elusive. Women with AMI are more inclined to gain a poorer outcome than men [1–3]. Plasma glucose is often considered as an important predictor of mortality after AMI [3–6]. But the impact of fasting plasma glucose (FPG) on early mortality and serious cardiovascular complications such as malignant arrhythmia, cardiac shock, heart failure, and reinfarction remains unclear.

Plasma glucose level in the acute phase of AMI is closely related to in-hospital mortality rate and serious complications of AMI. Previous studies have demonstrated that there is a near-linear positive relationship between admission plasma glucose (APG) or HbA1c and in-hospital mortality in diabetic and nondiabetic AMI patients [7, 8]. However, other studies showed that the relationship is u-shaped in AMI patients [9–11]. Moreover, previous studies mainly focused on the relationship between APG and clinical outcome. Limited data is available for association between FPG and clinical outcome of AMI with or without T2DM in female patients.

To assess the relationship between FPG and AMI prognosis in female patients, we conducted this retrospective analysis to determine the association between APG, FPG, and serious cardiovascular complications of female AMI patients with or without diabetes.

## 2. Patients and Methods

### 2.1. Subjects and Diagnostic Criteria

From January 2002 to February 2014, a total of 253 cases of consecutive female patients who were admitted to the Fifth Affiliated Hospital of Sun Yat-sen University in China with their first AMI diagnosis were enrolled into the retrospective study. AMI was defined by the following characteristics: chest pain consistent with ongoing myocardial ischemia persisting >30 minutes, ischemic electrocardiographic changes, and positive biochemical cardiac necrosis markers measurement (peak creatinine kinase value >2 times the normal upper limit or elevation of serum troponin I(cTnI) or serum troponin T(cTnT)). STEMI was diagnosed if ST-segment elevation ≥1 mm occurred in ≥1 lead or new left bundle branch block (LBBB) was found in ECG with biochemical evidence of myocardial necrosis. NSTEMI was diagnosed in patients with ≥1 positive biochemical cardiac necrosis markers measurement without new ST-segment elevation in ECG. Malignant arrhythmia is defined as fast or slow arrhythmia that significantly influences blood flow dynamics, including ventricular tachycardia, ventricular flutter, ventricular fibrillation, three-degree AVB, and fast atrial fibrillation with unstable hemodynamic. Cardiogenic shock was defined as reduced blood pressure (SBP < 90 mmHg or a drop of mean arterial pressure > 30 mmHg) and/or low urine output (<0.5 mL/kg/h), with a pulse rate > 60 bpm with or without evidence of organ congestion [12].

Enrolled patients were divided into diabetic group and nondiabetic group based on their final diagnosis [13]. Patients were thought to have diabetes if they had a previous or current diagnosis of diabetes, regardless of glycemic status on admission. The exclusion of T2DM was confirmed by the measurement of nonfasting glucose and fasting glucose before discharge. Each group was divided into two prespecified groups based on APG level (<11.1 and ≥ 11.1 mmol/L) and FPG level (<7.0 and ≥ 7.0 mmol/L). And we defined the former (APG < 11.1 mmol/L and FPG < 7.0 mmol/L) as nonhyperglycemia subgroup and the latter as hyperglycemia subgroup. This study excluded patients with a history of malignant tumor, chronic renal failure (creatinine > 451 *μ*mol/L), liver cirrhosis, serious infected diseases, and previous myocardial infarction.

### 2.2. Clinical Data Collection

Clinical symptoms and signs including chest pain or painless, systolic blood pressure (SBP), diastolic blood pressure (DBP), and heart rate (HR) were recorded on admission. Blood samples, including APG, creatinine, and creatinine kinase (CK), were measured at the time of hospital admission. FPG, total cholesterol (TC), triglyceride (TG), high-density lipoprotein cholesterol (HDL-C), and low-density lipoprotein cholesterol (LDL-C) were measured in the next day's morning after overnight fasting. According to the results of electrocardiograph (ECG), they were classified into non-ST-segment elevation myocardial infarction (NSTEMI) or ST-segment elevation myocardial infarction (STEMI). Killip class was used for the assessment of the severity of heart failure. The primary end point was all-cause in-hospital mortality; the second end points were serious cardiovascular complications such as malignant arrhythmia, cardiac shock, heart failure, and reinfarction.

### 2.3. Statistical Analysis

Continuous variables are expressed as mean ± SD or median ± interquartile range, and categorical variables were reported as numbers and percentages. Statistical analysis was performed with chi-square test for categorical variables. The* t*-test was used for continuous variables. Logistic regression analyses were used to determine the predictors of in-hospital mortality. In order to account for the influence of the risk factors on mortality rate, we performed a enter regression analysis with in-hospital death as outcome and the other risk factors (age, APG, FPG, SBP, DBP and HR at admission, blood lipid, and creatinine) as covariates. All 2-sided *P* values <0.05 were considered statistically significant. Analyses were done using the statistical software SPSS 13.0.

## 3. Results

### 3.1. Baseline Clinical Characteristics

From February 2002 to February 2014, a total of 253 female patients with their first AMI diagnosis were enrolled in this study. Diabetic group included 87 patients (34%) and nondiabetic group included 166 patients (66%). They had average ages of 70.11 ± 9.80 and 70.32 ± 12.30 years old, respectively. There was no difference in the age between the two groups, as shown in Figure 2.

Table 1 presents the baseline clinical characteristics of patients with or without diabetes. There was significant difference in APG and FPG between the two groups. Mean APG was significantly higher in diabetic patients than nondiabetic patients (13.9 ± 6.2 versus 8.5 ± 3.7 mmol/L, *P* < 0.01). Besides, mean FPG was also remarkably higher in diabetic patients (9.1 ± 3.5 versus 5.9 ± 1.3 mmol/L, *P* < 0.01). For the blood lipid profile, plasma triglyceride level was obviously higher and HDL-C was lower in diabetic group. Compared with the nondiabetic group, diabetic patients were more likely to have atypical clinical presentations of AMI. The proportions of painless AMI and NSTEMI were statistically higher among diabetic patients than the nondiabetic group. In addition, diabetic patients may have a greater reinfarction rate after the first AMI than nondiabetic patients (13.79% versus 6.02%, *P* < 0.05). However, the incidence rate of malignant arrhythmia in diabetes group was lower than nondiabetic group (2.3% versus 11.45%, *P* < 0.05).

### 3.2. Relationship of APG and FPG to Serious Complications of AMI

**Figure 1:**
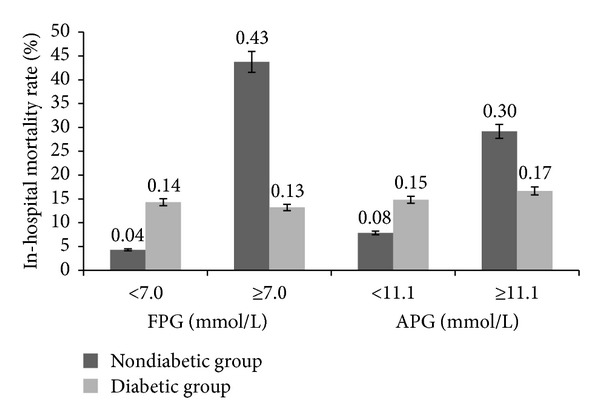
The relationship between blood glucose level and in-hospital mortality rate.

**Figure 2:**
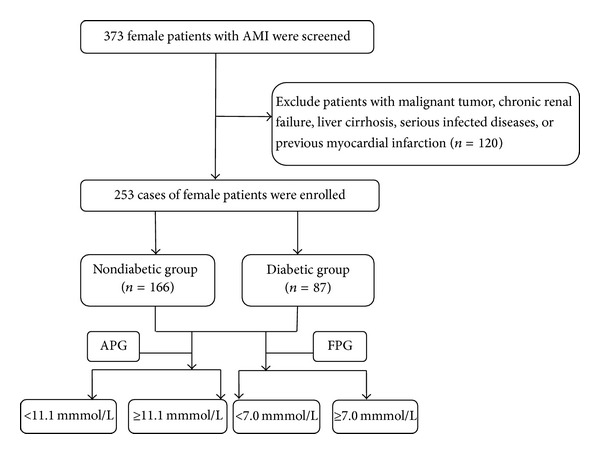
Flow chart of the inclusion of subjects in our study.

The association between different blood glucose level (APG and FPG) and clinical characteristics and complications of AMI was listed in Tables 2 (two missing values for FPG in non-diabetic group) and 3 (11 missing values for FPG in non-diabetic group and six in diabetic group). A total of 34 deaths (13.44%) occurred during hospital stay in two groups (Table 1). In-hospital mortality of diabetic group did not differ significantly from nondiabetic group (16.09% versus 12.05%, *P* = 0.37). But the mortality rate of nonhyperglycemia subgroup (APG < 11.1 mmol/L, FPG < 7.0 mmol/L) and hyperglycemia subgroup (APG ≥ 11.1 mmol/L, FPG ≥ 7.0 mmol/L) was dramatically different in the nondiabetic group (Tables 2 and 3). There was a tendency towards a much higher in-hospital mortality rate in nondiabetic group with the rising APG level and FPG level (Figure 1). When nondiabetic patients were subgrouped by APG level, the in-hospital mortality rate in nonhyperglycemia subgroup and hyperglycemia subgroup was 7.86% and 29.17%, respectively (*P* < 0.01). Likewise, when subgrouped by FPG level, the in-hospital mortality rate was 4.32% and 43.75%, respectively (*P* < 0.01). However, there was no significant difference between nonhyperglycemia subgroup and hyperglycemia subgroup in mortality rate in the diabetic group. Moreover, as shown in Table 3, in the nondiabetic group, the incidence of cardiac shock in hyperglycemia subgroup is much higher than nonhyperglycemia subgroup (12.95% versus 56.25%, *P* < 0.001).

### 3.3. Predictors of In-Hospital Mortality

In order to analyze which factor was closely associated with in-hospital mortality, we did a multivariate logistic regression analysis to eliminate the influence of confounding factors. Our results showed that independent predictors of in-hospital mortality for nondiabetic patients with AMI were FPG (OR: 2.014; 95% CI: 1.296–3.131, *P* < 0.01) and creatinine (OR: 1.011; 95% CI: 1.004–1.017, *P* < 0.01) (Table 4). However, the predictors of in-hospital mortality in diabetic group were age (OR: 1.160; 95% CI: 1.004–1.342, *P* < 0.05) and creatinine (OR: 1.007; 95% CI: 1.001–1.014, *P* < 0.05) (Table 5).

## 4. Discussion 

Several previous studies have demonstrated that elevated APG and HbA1c were powerful predictors of mortality and increased risk of cardiovascular complications in AMI patients both with and without diabetes [13–16]. They found that hyperglycemia was associated with larger infarct size [17], lower left ventricular ejection fraction (LVEF) [18], and poor prognosis. Previous reports also suggested that glycemic status, which poses a risk for CVD, differed in male and female individuals. The reason could be a difference in the basic mechanism of carbohydrate metabolism, hormone, and insulin sensitivity. But there were limited data on FPG and cardiovascular prognoses of female patients with AMI. In the present study, we defined fasting hyperglycemia in acute myocardial infarction phase to be ≥7.0 mmol/L and admission hyperglycemia to be ≥11.1 mmol/L. This glycaemia cutoff point was convergent with other authors' reports [19]. Moreover, this cutoff point was recommended by worldwide standards for a tool for carbohydrate metabolism disorder diagnosis [20]. Our study found that elevated FPG level in nondiabetic group was a strong and independent predictor of increased risk of mortality and cardiac shock in patients with AMI, and elevated APG level was closely associated with higher mortality rate and plasma creatinine level. But in diabetic group, we did not find this association. These results were in accordance with several previous studies including both men and women [21, 22].

In recent years, the importance of FPG in the prognosis of AMI was being gradually recognized. Suleiman et al. [21] reported a higher adjusted prevalence of in-hospital mortality among nondiabetic patients with elevated FPG, using a cutoff value of FPG > 6.1 mmol/L (110 mg/dL) for fasting hyperglycemia. Compared with patients categorized as having normal FPG, the adjusted OR for 30-day mortality progressively increased with higher tertiles of elevated FPG (first tertile: 4.6; 95% CI: 1.7 to 12.7; second tertile: 6.4; 95% CI: 2.5 to 16.6; and third tertile: 11.5; 95% CI: 4.7 to 20.0). Yang et al. [23] reported the FPG-stratified hazard ratios of in-hospital mortality in female patients of 1.037 (95% CI: 0.820–2.262) and 1.174 (95% CI: 0.905–4.432) in mildly hyperglycemic and severely hyperglycemic group after multivariate adjustment. In our present study, we also found the prognostic value of APG in female nondiabetic patients with AMI.

T2DM is already an established risk factor for patients with AMI [24–26]. However, in contrast to these findings, we found higher in-hospital mortality of AMI in nondiabetic patients, contrary to the existing knowledge that the mortality of AMI was common mainly in diabetics. Moreover, our study demonstrated that elevated FPG and APG levels in nondiabetic group were strong and independent predictors of increased risk of mortality with AMI, but, in diabetic group, they were not. It showed that risk factors other than DM had a stronger association with the mortality of AMI in these cases. These results were consistent with the findings in some studies [27, 28]. This phenomenon observed in the current study may be a result of several factors, including improved treatment in the acute phase of AMI and increased long-term survival resulting from aggressive secondary prevention in diabetic patients [27]. Besides, we found that the frequency of malignant arrhythmia in the nondiabetic group was much higher than diabetes group, suggesting that malignant arrhythmia may contribute to narrowing the gap of short-term mortality between nondiabetic and diabetic patients. However, the underlying mechanisms are still unclear. Previous research indicated that hypoglycemia caused an acquired long QT syndrome and prolonged cardiac repolarization causes fatal cardiac arrhythmias [29]. But it did not fully explain these results. Besides, our study showed that the frequency of myocardial reinfarction was obviously higher in diabetic group, which indicated adverse long-term outcome in diabetic patients. Some studies also found that T2DM may abolish the beneficial effect of primary PCI on long-term risk of clinical reinfarction [30, 31]. For diabetic patients, undergoing primary PCI had the similar reinfarction rate compared with those who received the thrombolysis treatment [31]. Therefore, although T2DM did not impact short-term mortality rate, it still influenced the long-term mortality rate of AMI patients.

The relationship of the cause and effect between hyperglycemia and in-hospital mortality of AMI is still uncertain. On the one hand, serious AMI can cause stress hyperglycemia, resulting from a surge of stress hormones such as adrenaline, noradrenalin, and cortisol which induce or exacerbate an insulin-resistant state [32]. Relative insulin deficiency and excess catecholamine reduced glucose uptake by the ischemic myocardium and promoted lipolysis which increased circulating free fatty acids [21]. On the other hand, hyperglycemia itself may lead to serious complications of AMI. Induction of endothelial dysfunction, oxidative stress, hypercoagulability, and impaired fibrinolysis may follow after hyperglycemia [21]. These factors ultimately produce vicious cycle. But some animal experiments indicated that short-term hyperglycemia may protest against ischemic myocardium [33, 34]. More researches will need to deeply uncover the mechanisms.

However, there are some reports contradicting our analysis and suggesting that the prognosis of diabetic patients may be significantly poor during the acute myocardial infarction phase. Moriyama et al. [35] presented a 2-fold higher hospital mortality rate in the subgroup of patients with DM and hyperglycemia in comparison to patients with DM without hyperglycemia. Likewise, Scuteri et al. [36] reported higher in-hospital mortality rate in the group of patients with DM and hyperglycemia. Multivariate analysis showed 5-fold risk of death in patients with hyperglycemia over 300 mg/dL and 2.8-fold in patients with hyperglycemia over 218 mg/dL, in comparison to patients with blood glucose level below 161 mg/dL.

In our analysis, the prognosis of patients without DM with concomitant hyperglycemia may, in part, be explained by the following differences in group characteristics. Firstly, patients with hyperglycemia were generally over 60 years, which agreed with a multivariate analysis showing that age was an independent factor of increased mortality rate in 1-year follow-up. In addition, nondiabetic patients with acute hyperglycemia showed an increased rate of inefficient fibrinolysis and the presence of multivessel coronary heart disease. These patients differed not only in the mentioned parameters but also in infarction severity including the concentration of myocardial necrotic markers and left ventricular ejection fraction from normal glucose patients. Hence, the importance of evaluation of plasma glucose during AMI for a better prognosis during follow-up period cannot be disregarded. However, we should realize that Blood glucose value (PFG and APG) is changeable in one day. Normally, the level of blood glucose changes within a limited range in non-diabetic patients. However, the inter- and intra-day glucose variability is relatively high in diabetic patients. In addition, blood glucose level is influenced by many other factors, such as diabetes status, serious complications and treatment. So before we analyse the association between hyperglycemia and complications of AMI, those factors should be took into consideration.

Several limitations of the research should be acknowledged. First, this was a single-center, nonrandomized, and retrospective study with a relatively small number of patients. Second, we did not evaluate long-term outcome of every AMI patient, so the relationship between FPG and long-term prognosis was not exactly assessed.

## Figures and Tables

**Table 1:** Baseline characteristics of patients with and without diabetes mellitus.

Variable	Nondiabetic group	Diabetic group	*P* value
Cases	166	87	
Age (years)	70.32 ± 12.30	70.11 ± 9.80	0.88
Hypertensions	98 (59.03%)	61 (70.11%)	0.08
Painless AMI	35 (21.08%)	30 (34.48%)	**0.02**
HR (bpm)	83 ± 23	86 ± 21	0.43
SBP (mmHg)	135 ± 30	140 ± 28	0.19
DBP (mmHg)	82 ± 20	80 ± 14	0.34
APG (mmol/L)	8.50 ± 3.73∗	13.90 ± 6.21	**0.00**
FPG (mmol/L)	5.90 ± 1.31^#^	9.10 ± 3.32^##^	**0.00**
CK (U/L)	719 ± 573	583 ± 560	0.36
TG (mmol/L)	1.26 ± 0.73	1.67 ± 0.98	**0.00**
TC (mmol/L)	5.20 ± 1.12	5.30 ± 1.40	0.63
HDL-C (mmol/L)	1.20 ± 0.34	1.09 ± 0.29	**0.01**
LDL-C (mmol/L)	3.11 ± 0.91	3.18 ± 1.08	0.63
Creatinine (*μ*mol/L)	92 ± 55	120 ± 106	0.08
NSTEMI	56 (33.73%)	41 (47.12%)	**0.04**
Conservative therapy	106 (63.86%)	63 (72.41%)	0.17
Primary PCI	55 (33.13%)	24 (27.59%)	0.37
Malignant arrhythmia	19 (11.45%)	2 (2.30%)	**0.01**
Cardiac shock	34 (20.48%)	15 (17.24%)	0.54
Killip classes III-IV	51 (30.72%)	33 (37.93%)	0.25
Mortality rate	20 (12.05%)	14 (16.09%)	0.37
Reinfarction rate	6.02%	13.79%	**0.04**
Reinfarction interval (month)	40 ± 21	18 ± 16	0.09

Data were presented as mean ± SD for normally distributed and continuous variables (Age, HR, SBP, DBP, APG, FPG, TC, HDL-C, and LDL-C) or median (IQR) for nonnormally distributed variables (CK, TG, creatinine, and reinfarction interval); categorical variables were reported as numbers and percentages.

AMI: acute myocardial infarction; HR: heart rate; SBP: systolic blood pressure; DBP: diastolic blood pressure; APG: admission plasma glucose; FPG: fasting plasma glucose; CK: creatinine kinase; TG: triglyceride; TC: total cholesterol; HDL-C: high-density lipoprotein cholesterol; LDL-C: low-density lipoprotein cholesterol; NSTEMI: non-ST-segment elevation myocardial infarction; and PCI: percutaneous coronary intervention.

∗Data for 164 patients, ^#^data for 155 patients, and ^##^data for 81 patients.

**Table 2:** Comparison of different admission glucose levels between nondiabetic group and diabetic group.

Variable	Nondiabetic group (mmol/L)		*P*	Diabetic group (mmol/L)		*P*
	<11.1	≥11.1		<11.1	≥11.1	
Cases	140	24		27	60	
Mortality rate	11 (7.86%)	7 (29.17%)	**0.006**	44 (14.81%)	10 (16.67%)	1.000
Painless AMI	27 (19.29%)	8 (33.33%)	0.121	6 (22.22%)	24 (40.00%)	0.107
TG	1.26 ± 0.81	1.12 ± 0.76	0.409	1.79 ± 1.21	1.62 ± 1.13	0.533
HDL-C	1.20 ± 0.34	1.20 ± 0.30	0.621	1.01 ± 0.25	1.13 ± 0.30	0.177
Creatinine	83.62 ± 57.20	96.04 ± 36.30	**0.013**	102.53 ± 77.26	97.5 ± 66.27	0.690
NSTEMI	47 (33.57%)	9 (37.50%)	0.708	13 (48.15%)	28 (46.67%)	0.898
Cardiac shock	24 (17.14%)	8 (33.33%)	0.116	3 (11.11%)	12 (20.00%)	0.479
Killip classes (III-IV)	38 (27.14%)	11 (45.83%)	0.065	9 (33.33%)	24 (40.00%)	0.553
Malignant arrhythmia	19 (13.57%)	5 (20.83%)	0.630	0 (0.00%)	2 (1.67%)	1.000

**Table 3:** Comparison of different fasting glucose levels between nondiabetic group and diabetic group.

Variable	Nondiabetic group (mmol/L)		*P*	Diabetic group (mmol/L)		*P*
	<7.0	≥7.0		<7.0	≥7.0	
Cases	139	16		28	53	
Mortality	6 (4.32%)	7 (43.75%)	**0.000**	4 (14.29%)	7 (13.21%)	1.000
Painless	29 (20.86%)	3 (18.75%)	1.000	7 (25.00%)	20 (37.74%)	0.248
TG	1.28 ± 0.79	0.98 ± 0.58	0.233	1.74 ± 1.21	1.65 ± 1.06	0.780
HDL-C	1.20 ± 0.34	1.16 ± 0.26	0.792	1.04 ± 0.26	1.12 ± 0.31	0.549
Creatinine	72.50 ± 37.25	63.00 ± 54.00	0.291	98.96 ± 72.25	100.02 ± 71.20	0.923
NSTEMI	48 (34.53%)	6 (37.5%)	0.813	12 (42.86%)	27 (50.94%)	0.488
Cardiac shock	18 (12.95%)	9 (56.25%)	**0.000**	2 (7.14%)	10 (18.87%)	0.278
Killip classes (III-IV)	34 (24.46%)	6 (37.50%)	0.408	11 (39.29%)	18 (33.96%)	0.625
Malignant arrhythmia	17 (12.23%)	3 (18.75%)	0.732	0 (0.00%)	1 (1.89%)	1.000

**Table 4:** Logistic regression analysis for mortality rate in nondiabetic group.

Variable	*B*	S.E.	Wald	*P*	OR	95% CI for OR	
						Lower	Upper
Age	0.057	0.031	3.286	0.070	1.059	0.995	1.126
HR	−0.014	0.015	0.834	0.361	0.986	0.957	1.016
SBP	−0.027	0.019	1.999	0.157	0.973	0.937	1.011
DBP	−0.004	0.030	0.020	0.886	0.996	0.938	1.056
APG	0.111	0.082	1.830	0.176	1.117	0.951	1.312
FPG	0.700	0.225	9.672	**0.002**	2.014	1.296	3.131
TG	−0.272	0.605	0.203	0.652	0.762	0.233	2.491
TC	0.050	0.674	0.006	0.940	1.052	0.281	3.939
HDL-C	−1.477	1.464	1.018	0.313	0.228	0.013	4.022
LDL-C	−0.097	0.769	0.016	0.900	0.908	0.201	4.096
CK	0.000	0.000	1.094	0.296	1.000	0.999	1.000
Creatinine	0.010	0.003	10.03	**0.002**	1.011	1.004	1.017

**Table 5:** Logistic regression analysis for mortality rate in diabetic group.

Variable	*B*	S.E.	Wald	*P*	OR	95% CI for OR	
						Lower	Upper
Age	0.149	0.074	4.044	**0.044**	1.160	1.004	1.342
HR	0.040	0.029	1.913	0.167	1.041	0.983	1.103
SBP	−0.064	0.035	3.422	0.064	0.938	0.876	1.004
DBP	−0.049	0.063	0.592	0.442	0.953	0.842	1.078
APG	0.122	0.090	1.827	0.176	1.129	0.947	1.347
FPG	0.025	0.139	0.032	0.858	1.025	0.781	1.346
TG	1.036	0.778	1.772	0.183	2.818	0.613	12.960
TC	−1.449	1.557	0.866	0.352	0.235	0.011	4.966
HDL-C	−0.237	2.890	0.007	0.935	0.789	0.003	227.501
LDL-C	1.749	1.575	1.233	0.267	5.748	0.262	125.928
CK	0.000	0.000	0.270	0.604	1.000	0.999	1.001
Creatinine	0.007	0.003	4.646	**0.031**	1.007	1.001	1.014
